# Ceftriaxone-resistant *Salmonella* Panama ST48 detected in poultry food chain: A phylogeographical analysis

**DOI:** 10.1016/j.onehlt.2025.101073

**Published:** 2025-05-12

**Authors:** Herrison Fontana, Bruna Fuga, Thais Martins-Gonçalves, Fernanda Esposito, Brenda Cardoso, Laura Rodrigues Beatriz, Nilton Lincopan

**Affiliations:** a Department of Clinical Analysis, School of Pharmaceutical Sciences, Universidade de São Paulo, São Paulo, Brazil; b One Health Brazilian Project (OneBR), São Paulo, Brazil; c Department of Microbiology, Institute of Biomedical Sciences, Universidade de São Paulo, São Paulo, Brazil; d Antimicrobial Resistance Institute of São Paulo (ARIES), São Paulo, Brazil; e Department of Celular Biology, Institute of Biological Sciences, University of Brasilia, Brasilia, Brazil; f Faculty of Agronomy and Veterinary Medicine, University of Passo Fundo, Passo Fundo, Brazil; g Department of Pathology, School of Veterinary Medicine and Animal Science, University of São Paulo, São Paulo, Brazil

**Keywords:** Nontyphoidal *Salmonella*, Foodborne pathogen, WHO critical-priority pathogens, One health, Genomic surveillance

## Abstract

*Salmonella* Panama is a clinically relevant serovar isolated from food sources, particularly poultry and swine, being also reported in human gastrointestinal and extraintestinal disease globally. The emergence of broad-spectrum cephalosporin-resistant *Salmonella* has been considered a One Health matter that demands continuous microbiologic and genomic surveillance for a comprehensive understanding and mitigation actions. During a local surveillance study conducted to monitoring WHO priority *Salmonella* spp. in the Brazilian poultry food chain, a ceftriaxone-resistant *Salmonella* strain (PN2) exhibiting a positive extended-spectrum beta-lactamase (ESBL) phenotype was recovered from a chicken sample, in a slaughterhouse under federal inspection. Clinically relevant genomic data confirmed *Salmonella* Panama sequence type (ST) 48 carrying the *bla*_CTX-M-8_ ESBL gene into the broad-host range IncM1conjugative plasmid, and displaying point mutations in the quinolone determining resistance region (QRDR), related to fluoroquinolone non-susceptibility. Phylogeographical analysis of publicly available global genomes of *S.* Panama ST48 revealed a potential concern for One Health issues due to its zoonotic nature, clustering PN2 (52–153 cgSNP differences) along with genomically related *S.* Panama strains isolated from human infections, food products, and farm animals, in the United States of America and Brazil. Interestingly, using Bayesian clustering method, the PN2 strain was grouped in the main clade C4, along with a CTX-M-55-producing *S.* Panama strain isolated from a human stool sample in Taiwan. In summary, we alert for the potential risk of dissemination of a neglected serovar that has contributed to the *Salmonella* global disease burden through the food supply chain.

## Highlights

• WHO critical priority *Salmonella* in the poultry food chain has been investigated.
• Ceftriaxone-resistant *S. panama* producing CTX-M-8 ESBL was identified.
• Genomic analysis revealed emergence of ST48 clone carrying IncM1/*bla*_CTX-M-8_ plasmid.
• Phylogeographical analysis of global *S. panama* ST48 revealed One Health distribution.
• Potential risk of dissemination of the emergent *S. panama* ST48 is discussed.

## Graphical abstract

**Figure:**
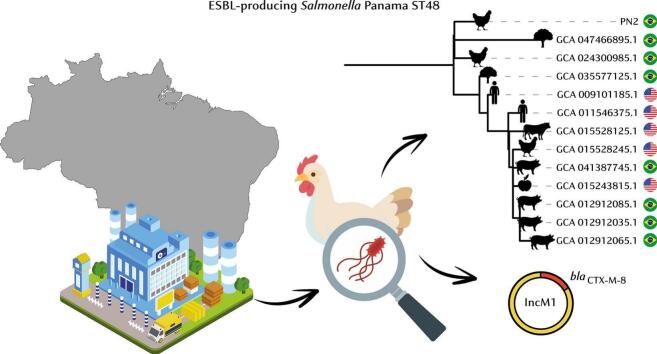

## 1 Introduction

*Salmonella* is one of the most clinically relevant zoonotic foodborne pathogens, being a public health threat worldwide [1,2]. Since ceftriaxone and ciprofloxacin are recommended for salmonellosis requiring antibiotic therapy, the development of antibiotic resistance is a cause for concern, limiting therapeutic options [3]. In fact, the updated list of bacterial priority pathogens, released in 2024 by the World Health Organization (WHO), has categorized third cephalosporin-resistant Enterobacterales and non-typhoidal *Salmonella* fluoroquinolone-resistant within the “critical” and “high” priority groups, respectively [4]. Therefore, the spread of plasmids encoding extended-spectrum beta-lactamase (ESBL) genes, which confers resistance to broad-spectrum cephalosporins, represents a pressing public health challenge that demands continuous surveillance [[1], [2], [3], [4]].

Although nontyphoidal *Salmonella* has emerged as a prominent pathogen in bloodstream infections, only a small group of serovars can cause systemic infection, known as invasive nontyphoidal salmonellosis. In this regard, *Salmonella enterica* serovar Panama is a serovar responsible for invasive salmonellosis worldwide, being reported in gastrointestinal and extraintestinal infections, such as septicemia, meningitis, and osteomyelitis [1,5]. Non-human sources of *S.* Panama include reptiles, seabirds, food-producing animals (i.e., poultry, swine, bovine), fresh produce, and environmental reservoirs [1,[6], [7], [8], [9]].

In this study, as part of the Grand Challenges Explorations: New Approaches to Characterize the Global Burden of Antimicrobial Resistance Program, we alert on the emergence of ceftriaxone-resistant *Salmonella* Panama ST48 clone bearing the transferable IncM1/*bla*_CTX-M-8_ plasmid in the poultry food chain.

## 2 Materials and methods

In 2013, during a local surveillance study conducted to monitor the presence of priority *Salmonella* spp., at slaughterhouse level, twenty samples were randomly collected from poultry in different batches. In this regard, one ceftriaxone-resistant *Salmonella* strain (designated PN2) was recovered from a cloacal swab collected in a slaughterhouse located in southern Brazil. Bacterial identification was accessed using MALDI-TOF/MS, whereas serotyping was performed using slide agglutination method and further confirmed by Premi®Test *Salmonella* PCR-based assay. Antimicrobial susceptibility testing was performed using the disc diffusion, and included amoxicillin-clavulanic acid (20/10 μg), azithromycin (10 μg), ceftriaxone (30 μg), ceftiofur (30 μg), ceftazidime (30 μg), cefepime (30 μg), ertapenem (10 μg), aztreonam (30 μg), amikacin (10 μg), gentamycin (10 μg), nalidixic acid (30 μg), ciprofloxacin (5 μg), enrofloxacin (5 μg), trimethoprim-sulfamethoxazole (1.25/23.75 μg), fosfomycin (30 μg), tetracycline (30 μg) and chloramphenicol (30 μg). Broth microdilution method was used to determine the minimum inhibitory concentration (MIC) for fluoroquinolones and colistin. The ESBL phenotype was screened using the double disc synergy test (DDST). Results were interpreted according to CLSI breakpoints [10].

Plasmid transferability was evaluated by conjugation using the kanamycin-resistant *Escherichia coli* C600 as recipient strain. Briefly, donor and recipient strains were inoculated in Luria broth (LB) at 1:1 ratio and incubated overnight at 37 °C. Transconjugants were selected on MacConkey agar plates supplemented with kanamycin (100 mg/L) and ceftriaxone (2 mg/L). The presence of the IncM1 plasmid, predicted by genomic analysis, was detected in transconjugants using PCR-based plasmid replicon typing [11], whereas DDST was conducted to evaluate successful conjugation of *bla*_CTX-M-8_/IncM1 plasmid.

Genomic DNA of strain PN2 was extracted from an overnight culture in LB broth using the PureLink™ Quick Gel Extraction Kit (Life Technologies, Carlsbad, USA) according to manufacturer's instructions. DNA quality and concentration were evaluated using Qubit 2.0 fluorometer (Life Technologies, Carlsbad, USA) and genomic libraries were prepared using a Nextera DNA Flex Library Preparation Kit (Illumina Inc. San Diego, CA). Afterwards, DNA sequencing was performed on an Illumina NextSeq550 platform using a paired-end protocol (2 × 75-bp). Short reads were quality checked and trimmed with TrimGalore v0.6.10 (https://github.com/FelixKrueger/TrimGalore) using PHRED33 and de novo assembly performed by Unicycler v3.15.5 (https://github.com/rrwick/Unicycler). Antimicrobial resistance genes, plasmid replicons and virulence genes were identified with Abricate v1.0.0 (https://github.com/tseemann/abricate) using the ResFinder, PlasmidFinder and VFDB databases, respectively. A minimum of 90 % threshold was applied for gene coverage and nucleotide identity. *Salmonella* pathogenicity islands (SPIs) were identified using the SPIFinder v2.0 tool hosted at the Center for Genomic Epidemiology (https://cge.food.dtu.dk). Serotyping was confirmed in silico with SeqSero2 v1.1.1 (http://www.denglab.info/SeqSero2).

To perform a phylogeographical and comparative genomic analysis, publicly available *S.* Panama genome assemblies, from both clonal and non-clonal isolates, were retrieved from the NCBI Pathogen Detection Database (Feb 20, 2025). All genomes were screened for multi-locus sequence type (ST) ST48 using MLST v2.23.0 (https://github.com/tseemann/mlst). Genome assemblies with missing metadata (i.e. geographical location, isolation source) were removed from dataset. Further, *S.* Panama PN2 strain (GenBank accession: GCA_043673855.1) and the resulting genome assemblies (*n* = 1098) were standard annotated by Prokka v1.12.8 (https://github.com/tseemann/prokka), and the core-genome determined with Roary v3.13 (https://github.com/sanger-pathogens/Roary). Single nucleotide polymorphisms of core genes (cgSNPs) were extracted using snp-sites (https://github.com/sanger-pathogens/snp-sites). Phylogenomic inference was performed by RAxML-NG v1.2.1 (https://github.com/amkozlov/raxml-ng) using the GTR + Gamma substitution model and 100 bootstraps. Tree topology, design and annotation was performed on iTol v6 (https://itol.embl.de). Bayesian analysis of population structure (BAPS) was performed using R package rhierbaps (https://github.com/gtonkinhill/rhierbaps). Default parameters were used for all software, unless otherwise specified.

## 3 Results and discussion

Nontyphoidal *Salmonella* species are among the most common enteric bacterial pathogen isolated from human infections [[1], [2], [3]]. Although most serovars cause uncomplicated gastroenteritis, and antimicrobial treatment is usually unnecessary, invasive infections requiring broad-spectrum antibiotics have emerged [3]. Broad-spectrum cephalosporins, especially ceftriaxone, are commonly used to treat invasive infections or severe diarrhea caused by *Salmonella* [3]. However, in recent years, ceftriaxone-resistant *Salmonella* has emerged in humans and livestock animals worldwide [12].

*Salmonella* Panama PN2 strain displayed resistance to clinically relevant antibiotics, including ceftriaxone, cefotaxime, cefepime, ceftiofur, aztreonam, whereas reduced susceptibility to ciprofloxacin (MIC, 0.5 mg/L) and enrofloxacin (MIC, 1 mg/L), while remained susceptible to carbapenems, aminoglycosides and colistin (MIC, 1 mg/L).

In silico serotyping and multi-locus sequence type prediction confirmed that ceftriaxone-resistant *Salmonella* PN2 belonged to serovar Panama (antigenic formula 9:l,v:1,5), and the international high-risk sequence type ST48. A total of nine pathogenicity islands (SPIs) and 93 virulence genes were identified in *Salmonella* Panama PN2 strain (Table S1), including fimbrial and non-fimbrial adherence factors, magnesium uptake, type III secretion system apparatus, resistance to antimicrobial peptides and the *cdtB* gene, which encodes a cytolethal distending toxin associated to host invasion [13], suppression of the host inflammatory response [14] and systemic spread [15].

Regarding resistome analysis, aminoglycoside acetyltransferase *aac(6′)-laa* chromosomal cryptic gene, *bla*_CTX-M-8_ and *bla*_TEM-1__A_ beta-lactamase genes and point mutations in *gyrA* (S83F) and *parC* (T57S) genes (Table S2), associated with fluoroquinolone resistance, were predicted. ESBL production was related to the presence of the CTX-M-8 gene, which was carried by the IncM1 plasmid, and was successfully transferred to *E. coli* C600 recipient strain via conjugation experiments.

The CTX-M-type ESBL enzymes are widely spread within Enterobacterales isolated from human infections and livestock globally, with the CTX-M-2, CTX-M-8, and CTX-M-15 ESBL variants being endemically in certain regions [16,17]. Therefore, the emergence of *Salmonella* Panama harboring the *bla*_CTX-M-8_ gene in poultry is particularly concerning, as it represents a potential risk for transmission of antimicrobial resistance thought the food chain.

The *bla*_CTX-M-8_ gene was first identified in the early 2000s, from clinical Enterobacterales, in Brazil [18], and since then have successfully spread across Asia, Europe, and South America in association with IncI1-pST113 and IncM1 conjugative plasmids [19]. Particularly, IncM1 plasmids bearing ESBL *bla*_CTX-M-8_ gene have been described among *Salmonella* serovars Enteritidis, Infantis and Newport, isolated from human clinical specimens [19].

*Salmonella* Panama has been reported as one of the most frequently isolated *Salmonella* serovar over the last years associated with invasive extraintestinal infections and foodborne outbreaks in USA and Europe [1]. In Brazil, *Salmonella* Panama is one of the most prevalent serovars isolated from human infections [20], being also isolated from wild birds, agriculture-impacted river, freshwater shrimp, retail meat products and along to the swine and poultry supply chain [[21], [22], [23]]. Currently, Brazil holds the second position of largest chicken producer in the world and is the main exporter, which the southern region accounts for over 78 % national production of chicken meat in 2023 [24]. In this country, the assurance of food quality and safety of animal derived meat products is supervised by the federal inspection system (SIF) that is under the Ministry of Agriculture, Livestock and Supply (MAPA) jurisdiction and exportations must be complying with sanitary regulation of the importing countries. Notably, Brazil exported 5139 mil ton of retail chicken meat in 2023 to China (13.61 %), United Arab Emirates (8.79 %), Japan (8.65 %). South Africa (6.79 %), Philippines (4.38 %) and European Union members (4.32 %), which imply a public health concern in the global spread of foodborne pathogens and antimicrobial resistance genes linked to Brazilian poultry production systems.

**Fig. 1:**
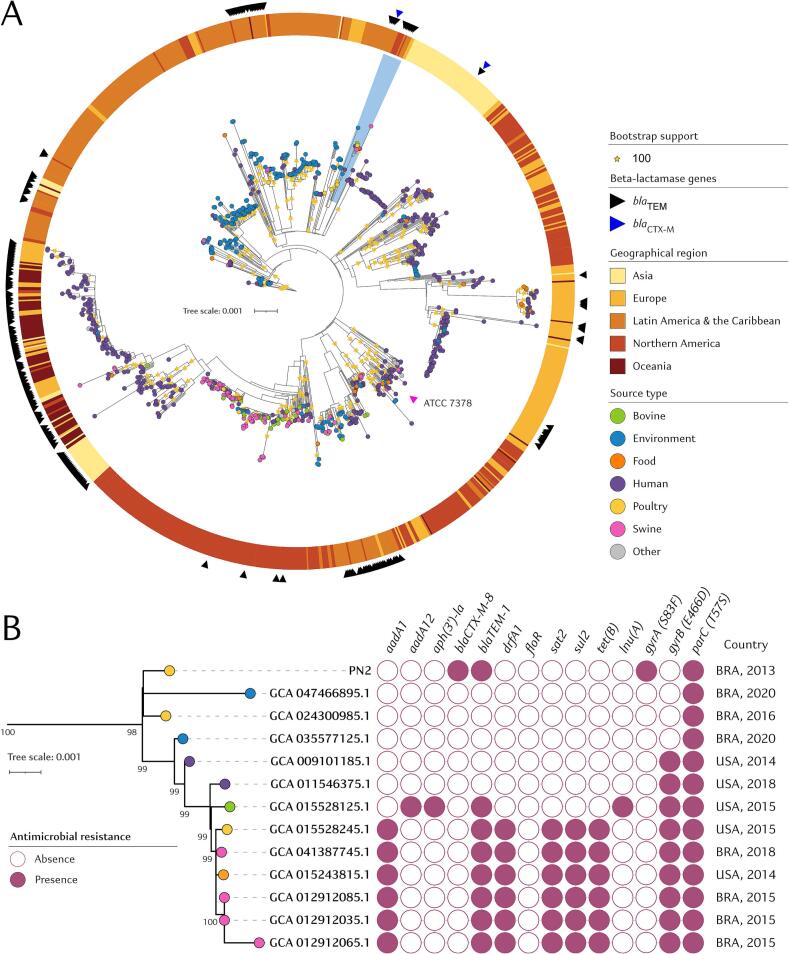
In A, maximum-likelihood phylogenetic tree of *Salmonella* Panama ST48 strains (*n* = 1098) publicly available genomes based on single nucleotide polymorphisms (cgSNPs) of 3750 core-genes. In B, pruned tree highlighting *Salmonella* Panama PN2 strain (this study, GenBank accession number: GCA_043673855.1) and related genomes isolated from livestock animals, food, environmental and human samples collected in United States and Brazil between 2013 and 2020. Country abbreviation follows the ISO 3166-1 alpha-3 code. Genome assembly associated metadata and cgSNP matrix are available in Table S2 and S3, respectively.

Previously studies on *Salmonella* spp. population structure have shown that *S.* Panama evolved from a common ancestor that originated *Salmonella* serovars Typhi, Sendai and Miami. Although *Salmonella* serovars Panama and Miami are clonally related and shares the ST48 and eBurstGroup 42, *S.* Panama presents a distinct phylogeny group [25,26]. Moreover, the interpretation of SNP distances in phylogenomic analyses based on core-genome is critical for understanding the genetic relatedness of bacterial pathogens. While *Salmonella* strains with fewer than 10 SNPs are often considered highly related and likely part of the same outbreak, strains with 10–50 SNPs are typically regarded as closely related, potentially sharing a recent common ancestor. It has been suggested that *Salmonella* serovars differing up to 200 SNPs may still belong to the same clonal lineage, even though not directly linked in time and space [27]. In this study, PN2 strain clustered to other *S.* Panama ST48 isolated between 2013 and 2020 from food-products, livestock animals, environmental and human clinical samples in Brazil and USA with 52–153 cgSNP differences (Table S2, Table S3) and bootstrap support values greater than 98 % (Fig. 1), indicating clonal relatedness within the Panama ST48 lineage. Interestingly, using Bayesian clustering method, PN2 strain was clustered in the main clade C4, along with a CTX-M-55-producing *Salmonella* Panama Sal-3071 strain isolated in 2015 from human stool sample in Taiwan (GenBank accession: GCA_007096325.1). However, even though both strains shared 169 cgSNP differences, they were grouped in different subclades within C4 clade. Moreover, PN2 and Sal-3071 strains harbored different plasmid incompatibility groups and ESBL variants from divergent CTX-M groups, suggesting independent pathways for the acquisition of resistance genes.

**Fig. 2:**
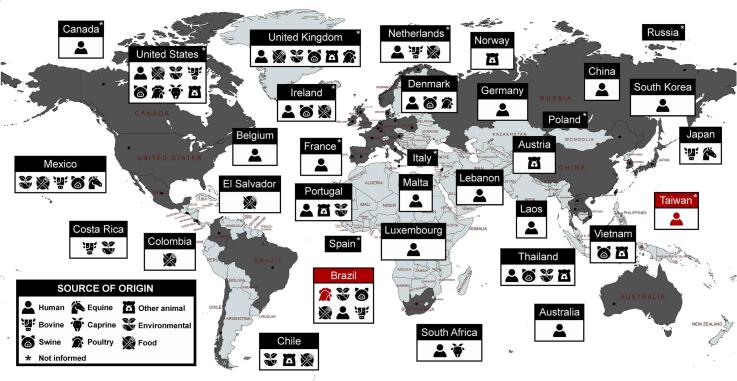
Map of geographical distribution and isolation source of *Salmonella* Panama ST48 circulating at the One Health interface worldwide (1989–2023). CTX-M-producing *Salmonella* Panama ST48 are highlighted in red. Data were retrieved from the NCBI Refseq database (http://www.ncbi.nlm.nih.gov/RefSeq/) and EnteroBase (https://enterobase.warwick.ac.uk/) available in Table S2 and S4. (For interpretation of the references to colour in this figure legend, the reader is referred to the web version of this article.)

Our comparative analysis highlights global spread of *Salmonella* Panama ST48 strains in countries from the Americas, Asia, Europe and Oceania (Fig. 2, Table S4) harboring multiple resistance genes against clinically important antimicrobials, including beta-lactams (*bla*_CTX-M-55_, *bla*_TEM-1__A_, *bla*_TEM-1B_), quinolones (*oqxAB, qnrB2*, *qnrB19*, *qnrS1*), macrolides [*mph*(A), *ere*(A), *erm*(T)], fosfomycin (*fosA3*), colistin (*mcr-9*, *mcr-1*) phenicol (*catA1*, *cmlA1*, *floR*), tetracycline [*tet*(A), *tet*(B), *tet*(D), *tet*(M)], folate pathway inhibitors (*sul1*, *sul2*, *sul3*, *drfA*) and aminoglycosides (Table S2).

In summary, we report the emergence of critical priority *S.* Panama ST48, an emerging clone widely disseminated among farmed animals, including poultry, swine, and bovine, and causing human infections; alerting for potential risk of dissemination of a neglected serovar that has contribute to the *Salmonella* global disease burden through the food supply chain.

## CRediT authorship contribution statement

**Herrison Fontana:** Writing – review & editing, Writing – original draft, Visualization, Methodology, Investigation, Formal analysis, Data curation, Conceptualization. **Bruna Fuga:** Writing – review & editing, Writing – original draft, Methodology, Investigation, Formal analysis, Data curation, Conceptualization. **Thais Martins-Gonçalves:** Writing – review & editing, Writing – original draft, Visualization, Methodology, Investigation, Formal analysis, Data curation. **Fernanda Esposito:** Writing – review & editing, Writing – original draft, Methodology, Formal analysis, Data curation. **Brenda Cardoso:** Writing – review & editing, Writing – original draft, Methodology, Formal analysis, Data curation. **Laura Rodrigues Beatriz:** Writing – review & editing, Writing – original draft, Investigation. **Nilton Lincopan:** Writing – review & editing, Writing – original draft, Supervision, Methodology, Investigation, Funding acquisition, Formal analysis, Data curation, Conceptualization.

## Funding statement

This study was supported by 10.13039/100000865Bill & Melinda Gates Foundation (Grand Challenges Explorations Brazil (Grant OPP1193112), 10.13039/501100001807Fundação de Amparo à Pesquisa do Estado de São Paulo (FAPESP, Grant 2020/08224–9), 10.13039/501100003593Conselho Nacional de Desenvolvimento Científico e Tecnológico (CNPq, Grant 422984/2021–3), and 10.13039/501100002322Coordenação de Aperfeiçoamento de Pessoal de Nível Superior (CAPES, Fellowships Grants 88887.506496/2020-00 and, 88882.333054/2019-01). H.F is a research fellow of 10.13039/501100001807FAPESP (Grant 2024/19006-3). T.M-G is a research fellow of FAPESP (Grant 2024/20180-8). F.E is a research fellow of FAPESP (Grant 2019/15578-4). N.L. is a research fellow of 10.13039/501100003593CNPq (Grant 314336/2021-4).

## Declaration of competing interest

The authors declare no competing financial interest or personal relationship that could have appeared to influence the work reported in this paper.

## Data Availability

This whole-genome shotgun project has been deposited at DDBJ/ENA/GenBank under the accession number SAMN41107065. Additionally, genomic data of *Salmonella enterica* subsp. *enterica* serovar Panama PN2 strain is also available on the OneBR platform under the ID number ONE446 (http://onehealthbr.com/).

## Appendix A Supplementary data

Supplementary material 1 Image 1

Supplementary material 2 Image 2

Supplementary material 3 Image 3

Supplementary material 4 Image 4
